# Bacteriophages and Their Enzymes: Allies Against Microbial Biofilms

**DOI:** 10.3390/ph18121771

**Published:** 2025-11-21

**Authors:** Fohad Mabood Husain, Andaleeb Zahra, Asghar Ali, Mohan Kamthan, Nasser A. Al-Shabib, Zeba Farooqui, Naved Ahmad, Thamer Albalawi, Pravej Alam, Nayla Munawar

**Affiliations:** 1 Department of Food Science and Nutrition, College of Food and Agriculture Sciences, King Saud University, Riyadh 11451, Saudi Arabia; fhusain@ksu.edu.sa (F.M.H.); nalshabib@ksu.edu.sa (N.A.A.-S.); 2 Department of Botany, School of Chemical and Life Sciences, Jamia Hamdard, New Delhi 110062, India; 3 Clinical Biochemistry Lab, Department of Biochemistry, School of Chemical and Life Sciences, Jamia Hamdard, New Delhi 110062, India; mohan.kamthan@jamiahamdard.ac.in; 4 Department of Biomedical Engineering, College of Medicine, University of Illinois, Chicago, IL 60607, USA; zeba25@uic.edu; 5 Department of Computer Science and Information System, College of Applied Sciences, AlMaarefa University, Riyadh 13713, Saudi Arabia; nahmad@um.edu.sa; 6 Department of Biology, College of Science and Humanities, Prince Sattam Bin Abdulaziz University, Alkharj 11942, Saudi Arabia; t.albalawi@psau.edu.sa (T.A.); alamprez@gmail.com (P.A.); 7 Department of Chemistry, College of Science, United Arab Emirates University, Al-Ain 15551, United Arab Emirates

**Keywords:** antibiotic resistance, biofilms, endolysins, depolymerases, microbial communities, bacteriophage-derived enzymes

## Abstract

Bacterial biofilms pose a substantial challenge in healthcare and industrial and environmental settings because of their resilience and antibiotic resistance. Biofilm formation is a complex process involving microbial communities encased in an extracellular matrix that contributes to increased resistance and persistent infections. This review explores the emerging roles of bacteriophages and their derived enzymes as promising alternatives or adjunct therapies to combat bacterial biofilms. Bacteriophages, viruses that infect bacteria, exhibit marked specificity and diverse mechanisms for targeting and lysing bacterial cells within biofilms. Enzymes, including endolysins and depolymerases, have demonstrated efficacy in disrupting biofilm matrices. Moreover, the potential synergy between bacteriophages and antibiotics enhances their antimicrobial activity, providing a multifaceted approach for combating biofilm-associated infections. This review critically evaluates the current research, highlighting the successes and limitations of bacteriophage-based strategies in biofilm control, and underscores the potential of these alternatives in shaping future therapeutic interventions against biofilm-related bacterial infections.

## 1. Introduction

The ongoing emergence and spread of drug resistance in microbes has made infectious diseases a major cause of global morbidity and mortality in humans [1]. The evolution of drug-resistant strains and the re-emergence of old resistant microbial pathogens are major challenges for public health management [2]. The discovery of antibiotics is one of the most notable discoveries in modern medicine and has led to the cure of numerous infectious diseases. However, the unrestricted and extensive use of antibiotics has imposed selective pressure on bacteria, enabling them to develop antimicrobial resistance (AMR) [3,4,5]. Horizontal gene transfer in bacteria is an evolutionarily conserved strategy that worsens AMR. The development of AMR is recognized by the World Health Organization (WHO) as one of the greatest threats to the public regarding the management of infectious diseases [6,7]. To attract international attention to the escalating severity of AMR, in 2011 WHO celebrated World Health Day with the theme, “Antimicrobial resistance: no action today, no cure tomorrow” [8]. The microbes have developed resistance even against the recently developed antibiotics, such linezolid, vancomycin, and the latest β-lactams, and these antibiotics have lost their efficacy against some of the bacterial strains, such as vancomycin-resistant *Enterococci*, methicillin resistant *Staphylococcus aureus* (MRSA), drug-resistant *Salmonella* and *Acinetobacter* species [6,9,10]. Ceftaroline, a broad-spectrum antibiotic, is active against MRSA. It was approved for use by the Food and Drug Administration in 2010. This fifth-generation cephalosporin drug is expected to treat several infections caused by Gram-positive cocci; however, several reports of MRSA strains showing decreased susceptibility to this drug are emerging [11,12].

Thus, the discovery of new antibiotics has substantially decreased. Moreover, the recently developed drugs are strictly reserved for treating infections caused by extensively drug-resistant microbes [13]. However, the introduction and development of combination therapies has yielded promising results. It has been somewhat successful in improving the efficacy of antibiotics, while simultaneously limiting the chances of AMR development [14].

To date, most antimicrobials used for the treatment of bacterial infections, food preservation, and environmental settings aim to kill or inhibit microbial growth. An obvious drawback of this strategy is the creation of selective pressure, which leads to AMR [8,15]. The most common groups of problematic multi-drug resistant (MDR) bacteria are MRSA, *Mycobacterium tuberculosis*, vancomycin-resistant *Escherichia coli*, ESβL-producing MDR enteric bacteria, and *Pseudomonas aeruginosa.* These factors are responsible for innumerable deaths worldwide. Considering these issues, there is an urgent need to develop new chemotherapeutic antimicrobial compounds and identify novel methods for the prevention and treatment of infectious diseases. The development of new alternative treatment strategies, identification of novel drug targets, and development of cognate pharmaceutically applicable drugs capable of providing sustainable and long-term effectiveness against bacterial pathogens are required.

One emerging approach is the targeting of bacterial biofilms. Biofilms are formed by multiplying bacteria, which form communities that adhere to each other and surfaces, and play a crucial role in the persistence of bacterial infections [16]. Bacteria secrete a polysaccharide matrix, lipoproteins, fibrin, and numerous other substances to form extracellular polymers that are enclosed in this self-produced polymeric matrix to form biofilms. These natural polymers are crucial for biofilm structural stability and integrity [17]. The polymer matrix serves as a barrier, preventing the diffusion of disinfectants and antibacterial agents into the biofilm [18]. Most microbes can form biofilms, and once the biofilm is established, it overpowers the host immune system, thereby improving the survival rate of bacteria. Biofilm growth protects microbes from harsh environmental conditions and persistent infections [19]. Biofilms play an important role in nosocomial infections, particularly in immunocompromised patients [20]. Biofilms formed on medical devices or wounds are likely to result in successful infections. Most nosocomial infections are attributed to biofilms that develop on indwelling devices, such as cardiac pacemakers, catheters, and dentures. These surfaces are ideal for microbial attachment and biofilm formation [21]. As the immunity weakens, bacteria are released from the biofilm to establish infections at new sites. These biofilms allow bacteria to persist under pathological conditions, leading to chronic infections [22]. Biofilms also affect the virulence and viability of bacteria, not only in clinical settings but also in food industries that require a hygienic environment, especially during food processing and preservation [23,24]. Biofilms have been found on many surfaces, such as wood, stainless steel, polyethene, glass, and rubber, which enhance their presence [25]. However, the disadvantages of using biofilms in the food industry are not limited to their pathogenicity. It also results in the corrosion of metal surfaces and changes in organoleptic properties owing to the secretion of proteases and lipases. Such effects are important in food-based industries because various processes and structures serve as surface substrates for biofilm development at different temperatures for different microbial species [26]. Moreover, genes associated with biofilm formation may also have genomic variations, leading to the establishment of completely different biofilms under different sets of conditions. These factors render the eradication of biofilms a Herculean task [27]. Thus, there is an urgent need to develop alternative therapies to combat drug resistance in bacteria, particularly those associated with pathogenic biofilms.

Phages can be used as alternatives to drugs. They are abundant in the natural environment, where they can be isolated. They are natural predators of bacteria and represent an effective strategy against bacterial biofilms [28]. Bacteriophages were initially described by Felix d’Herrelle and Frederick Twort. These viruses infect bacterial hosts. Host range refers to the bacterial species on which phages can act. The host range can be narrow or broad. The phage preference is determined by the bacterial isolates that supported its replication [29]. Many studies have demonstrated the ability of phages to effectively infect and lyse bacterial cells present within the exopolysaccharides (EPS) as mono- or mixed-species biofilms [30,31]. They can enzymatically break down the biofilm matrix and enhance the local activity in association with biofilms [32]. This finding supports the concept of phage therapy and provides a complementary or alternative strategy for controlling biofilm-associated infections. The interaction between phages and biofilms is complex. Limited information is available on the overall effect of phages on biofilms and how phages interact with different bacterial populations in biofilms. This review aims to compile the current knowledge on phage-biofilm associations and possible phage-based strategies, including the synergistic action of antibiotics to overcome pathogenic biofilms.

## 2. Characterization of Bacterial Biofilms

### 2.1. Biofilm Formation

Biofilm development is broadly a three-stage process: the attachment stage, followed by the formation of microcolonies and maturation of biofilms. The dispersal of mature, differentiated biofilms occurs through mechanical and active processes [33]. The initial adhesion of microbes is governed by Lifshitz-Van der Waals, acid-base, electrostatic, and hydrophobic interactions [34]. Some surface-associated proteins, such as fibronectin-binding proteins, OmpA, protein A, biofilm-associated proteins, and SasG, are important for biofilm formation during the initial stages [35]. The process of quorum sensing, a cell density-dependent bacterial communication system, also plays a vital role in bacterial colonization and biofilm formation [20].

The second stage of biofilm formation is irreversible and begins with EPS secretion. This process continues until the bacteria completely attach to the surface inside the complex matrix [36]. Mature biofilms are complex three-dimensional structures comprising channels responsible for the transport of nutrients and water, along with small cavities to house planktonic bacteria. The structure and organization of biofilms differ between microbes; however, the exact underlying reason for their differentiation is unknown. For instance, biofilm formation in *Pseudomonas putida* is governed by LapA, an adhesion protein, while in other pseudomonads, such as *P. aeruginosa*, the ESPs, Pel and Psl, control biofilm formation [37,38,39]. The differences in matrix components may be responsible for the differences in biofilm structure. Finally, the established biofilms are eroded or sloughed off to release the bacteria into the environment [40].

The role of c-di-GMP, an intercellular secondary messenger, in the initiation of biofilm formation and virulence has been established [39,41,42]. c-di-GMP binds to various receptors, such as adaptor proteins, enzymes, riboswitches, and transcription factors [43]. Certain transducer mechanisms and environmental factors increase the c-di-GMP levels in bacterial cells. Increased c-di-GMP levels initiate adhesin production and play a key role in the secretion of the extracellular matrix [44,45]. Production of CdrA adhesion, alginate Pel, and Psl in *P. aeruginosa* is positively regulated by c-di-GMP molecules [46,47]. In addition to c-di-GMP, small regulatory RNAs (sRNAs) also play an important role in biofilm formation by several microbial species [48].

### 2.2. Antibiotic Resistance in Biofilms

The antibiotic resistance of bacteria residing in biofilms facilitate serious, persistent infections, and it is estimated that biofilms are responsible for more than two-thirds of all chronic infections. Bacteria present in biofilms has approximately 1000 times greater resistance to antibiotics than free-living cells; therefore, conventional drugs have proven inadequate for treating biofilm-based infections [49,50].

Multiple factors, based on the different molecular mechanisms of bacterial cell defense, contribute to the resistance of biofilms to antibiotics. Microorganisms in biofilms exhibit both intrinsic and acquired resistance mechanisms. According to previous reports, the resistance of biofilms can be attributed to: (A) the interaction of antibiotics with the biofilm matrix that impedes their action, (B) retarded growth of the bacteria within biofilms making the antibiotics ineffective, (C) genetic changes in the pathogens, (D) production of persister cells, (E) modification of the chemical microenvironment, (F) presence of multiple bacterial species, and (G) age of the biofilm [51,52].

The biofilm matrix is vital for bacterial resistance, as it shields cells from various physical, chemical, and biological stressors and acts as a barrier to the spread of antibiotics through the matrix [51]. The impermeable nature of the biofilm matrix, along with the presence of multiple bacterial species in the biofilms, renders antibiotics ineffective. Antibiotics have proven effective in reducing biofilms, but have failed to eradicate them [21,51].

The EPS matrix acts as an effective barrier to prevent the diffusion of antibiotics into the biofilms [53]. Alginate, a key component of the EPS matrix, blocks the diffusion of gentamicin or tobramycin, and EPSs nullify the action of aminoglycosides by directly binding to the cationic group of antibiotics and protecting *P. aeruginosa* biofilms [54,55,56]. Furthermore, EPSs play a key role in holding cells together and allow the consortia of different cells to function as multicellular systems. A density-dependent cell–cell communication system, QS, often regulates biofilm formation [17,57]. Biofilm formation is typically a QS-regulated phenotype [58]. Bacterial cells residing in biofilms are enclosed in an extracellular matrix comprising a mixture of various biological polymers, such as polysaccharides, proteins, nucleic acids, and lipids. This natural matrix protects cells from harsh environmental conditions and confers drug resistance by limiting the entry of antibiotics, and resists attacks of the host’s immune system [18]. The motility of bacteria and cell-to-cell communication play extremely important roles in biofilm formation, maintenance, and development of resistance. Chua et al. observed the role of QS in biofilm formation [59]. They reported the development of colistin-tolerant subpopulations in *P. aeruginosa* biofilms. Notably, cells of this subpopulation could move towards dead antibiotic-treated biofilm cells and initiate the formation of new biofilms using QS. In another study, components of QS systems (fsrA, fsrC, and gelE) were responsible for biofilm formation by antibiotic-treated *Enterococcus faecalis* [60]. Similarly, decreased biofilm formation by *S. aureus* has been observed upon inhibition of the QS system. Moreover, the biofilms became more susceptible to the action of different classes of antibiotics [61].

Persister cells are a group of slow-growing or growth-arrested bacteria that develop because of relatively poor diffusion of nutrients and oxygen in the periphery of biofilms. They are highly tolerant to the action of antibiotics, and their resistance is not genetic [62]. Although persister cells account for a very small portion (0.1–10%) of the entire biofilm population, these cells are capable of surviving 1000 times the minimum inhibitory concentration (MICs) of different antibiotics [63].

Another mechanism that appears to be responsible for the antibiotic resistance of biofilms is the presence of cells that possess resistance genes encoding enzymes that can render antibiotics ineffective. Enzymes, such as β-lactamases and aminoglycoside adenylyltransferases, are secreted in the biofilm matrix and prevent the antibiotics from reaching their target cells [53,64]. β-lactamases secreted by *Klebsiella pneumoniae* biofilm were observed to be effective in degrading ampicillin and blocking it from reaching and acting on target cells in the biofilm [65]. Young biofilms of *P. aeruginosa* are more susceptible to the actions of ceftazidime and meropenem than mature biofilms. This increased resistance of mature biofilms may be due to the presence of increased levels of β-lactamases in the matrix [66].

Oxygen limitation in the peripheral and lower layers of the biofilm structure may also account for increased antibiotic resistance in bacteria. Due to limited oxygen availability, most metabolic activity is confined to a narrow zone near the air interface, and bacteria in biofilms outside this zone are not easily eradicated by antibiotics [67]. For instance, oxygen limitation was correlated with increased ciprofloxacin and tobramycin tolerance in *P. aeruginosa* biofilms [68]. Similarly, decreased nutrient availability is another factor that contributes to increased resistance of biofilm cells. There is a well-established positive relationship between the bacterial growth rate and bactericidal antibiotic efficacy. Thus, cells with reduced nutrient supply demonstrate lower levels of metabolic activity, reduced growth rate and survival in a dormant state, and increased antibiotic tolerance [69]. Such metabolically repressed antibiotic-tolerant cells have been recovered in biofilms of *P. aeruginosa* in vitro as well as in the sputum of patients with cystic fibrosis [20,70].

Most of the biofilms formed in nature comprise different bacterial species, meaning that the biofilms are multi-species. Notably, multi-species biofilms demonstrate enhanced resistance to antibiotics compared with that of monospecific biofilms because of cooperative interactions between different bacterial species. This increased resistance is believed to be a consequence of increased biomass and/or altered composition of the EPS matrix [71]. Furthermore, the age of the biofilm is assumed to be important for the effectiveness of antibiotics. Mature biofilms are difficult to eradicate, possibly due to differences in their structure, EPS composition, and/or phenotypic changes [72].

Most chemical disinfectants and antibiotics are effective against free-living bacteria, ineffective when used against bacteria in a biofilm. Furthermore, the development of new antibiotics is slow and complex [35,73]. These reasons promote the use of bacteriophages to treat biofilms.

## 3. Bacteriophages as Inhibitors of Bacterial Biofilm

Bacteriophages are widespread in all habitats where their hosts are present, with an estimated range of 10^31^ to 10^32^ phages [74]. They are 10 times more abundant than bacteria in nature [75]. The wide diversity of phages is due to their dynamic adaptability to selective pressures. Bacteriophages are classified on the basis of their shape, size, and nucleic acid type [75,76]. Phages can be broadly classified as tailed, polyhedral, filamentous, and pleomorphic phages based on their basic structure [77]. Bacteriophages display host specificity owing to tail spike proteins that recognize specific ligands on the host surface during the adsorption stage. Notably, on a few occasions, bacteriophages only target a particular serotype of bacterium. This implies that non-pathogenic, untargeted bacteria remain unaffected by phage-mediated treatment [78]. However, the polyvalent nature of bacteriophages is well known, as they can target different strains of the same or different species [79].

**Figure 1:**
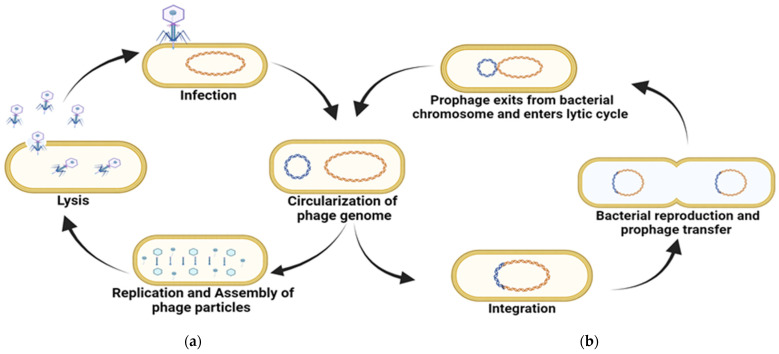
Diagrammatic representation of (**a**) lytic cycle and (**b**) lysogenic life cycles.

Bacteriophages infect bacteria during their lytic and lysogenic life cycles (Figure 1). Lytic phages lyse the host cells. They attach to receptors on the surface of bacteria, inject their genomic content into it, utilize the host machinery to manufacture their progeny by replication in the cytoplasm, and release them from the host cell into the environment. The new bacteriophages then repeat this process. The antibacterial potential of bacteriophages is often linked to their lytic forms, because the bacterial host is expected to die. In contrast, after ingestion, the lysogenic phage genome integrates with the host chromosome (prophage) to achieve a coexisting stage and is transferred to progeny cells until the lytic cycle is triggered by environmental factors, leading to the death of a part of the infected population. Bacteria infected with lysogenic phages are resistant to infections with related phages [80,81].

Most chemical disinfectants and antibiotics are effective against free-living bacteria but are ineffective when used against bacteria growing in a biofilm. Furthermore, the development of new antibiotics is slow and complex [35,73]. Recently, the scientific community has shown great interest in phage therapy as a substitute for conventional bactericidal drugs that target bacterial biofilms [81]. Phage particles alone, a cocktail of phages, phage proteins, enzymes, and a combination of phages and antibiotics have been reported as alternatives for controlling biofilm infection [80,81,82].

### 3.1. Use of Phages Against Biofilm-Forming Bacteria

Phages offer a promising alternative to antibiotics for the removal of harmful bacteria and their biofilms, with immense potential for medical and environmental applications. *P. aeruginosa*, an opportunistic nosocomial pathogen, forms stable, resistant biofilms. WHO has designated it as a key priority in the development of novel therapeutics. Two phages isolated from wastewater plants, Podovirus (φMR299-2) and myovirus (φNH4), eliminate *P. aeruginosa* biofilm on a pulmonary cell line [83]. Phage P100 was evaluated for its antibiofilm potential against 21 strains of the food-borne pathogen, *Listeria monocytogenes*. P100 significantly impaired biofilm formation in *L. monocytogenes* irrespective of serotype, growth conditions, or biofilm-forming capabilities. Phage treatment was effective even on the biofilm formed on stainless steel and resulted in 3.5–5.4 log/cm^2^ reduction in biofilm [84]. Similarly, the efficacy of the three phages, LiMN4L, LiMN4p, and LiMN17, in reducing biofilms of *L. monocytogenes* were assessed. Treatment with these phages (10^9^ pfu/mL) resulted in 3 3-log unit decrease in bacterial adhesion [85]. Lytic phage EFDG1 effectively killed planktonic and biofilm cells of *E. faecalis* both in vitro and in vivo [86]. The three lytic phages significantly reduced the crystalline biofilm of the uropathogen, *Proteus mirabilis*, formed on the catheter. It causes severe infections by forming dense biofilms [87]. The presence of *K. pneumoniae* biofilm caused prosthetic reinfections in a patient who underwent total knee arthroplasty. Intravenous application of phage KpJH46φ2 resulted in decreased biofilm biomass of *K. pneumoniae* after 22 h [88].

### 3.2. Phage Cocktail Therapy

Another phage-based therapy that has shown substantial potential for treating persistent bacterial infections is phage cocktail therapy. Phages with different host ranges and target receptors combine to form a phage cocktail that possesses broad-spectrum activity, hindering the development of phage-resistant bacteria. A cocktail of *E. coli* O157:H7-specific bacteriophages was shown to inhibit a 48 h biofilm formation on spinach harvester blades and reduce the viable counts of adhered *E. coli* cells by 4.5 log units after 2 h of treatment [89]. Another study reported that the cocktails composed of three and six phages were effective in reducing the biofilm of Shiga toxin-producing *E. coli* (STEC) by 47.04 and 48.35%, respectively [90].

*S. aureus* is an important human and food pathogen, which forms calcitrant biofilms on almost all types of surfaces. Staphylococcal phage K, along with a cocktail of derivative phages, reduced biofilm formation in *S. aureus* [91]. Additionally, a mixture of phage K and DRA88 effectively lysed three *S. aureus* strains; diminished biofilm formation was observed after 4 h of phage treatment and complete removal was recorded after 48 h [92]. In a recent study, seven commercially available phage cocktails were assessed for their biofilm-inhibitory potential against *S. aureus* isolated from patients undergoing peritoneal dialysis. Six of the seven phage cocktails demonstrated promising activity against the biofilms formed by the isolated *S. aureus* strains, and the resistance of the *S. aureus* strains to the phages was negated by adaptation [93].

A cocktail of three phages, LiMN4L, LiMN4p, and LiMN17, was tested against 7-day biofilms formed by three *L. monocytogenes* strains on stainless-steel coupons, and the cell counts were reduced to undetectable levels after 75 min [85].

The effects of six *P. aeruginosa* and four *P. mirabilis* phage mixtures were evaluated against the single and dual species biofilm of *P. aeruginosa* and *P. mirabilis*. The cocktail treatment reduced *P. aeruginosa* and *P. mirabilis* biofilm counts by 4 log CFU/cm^2^ and >2 log CFU/cm^2^, respectively [30]. Six novel lytic phages were selected to prepare a cocktail with the potential to inhibit biofilm formation by *P. aeruginosa* PAO1. Biofilm formation was studied under static and flow conditions, and the cocktail was found to disperse and eliminate the biofilm biomass under both sets of conditions. Under static conditions, almost a 95% reduction in the biofilm of PAO1 was recorded after 4 h of phage application, while under flowing conditions, although the activity was slow but still after 48 h of phage treatment, almost all cells were removed, and the biofilm was dispersed [94]. In a similar study, a mixture of three phages isolated from hospital wastewater demonstrated significant biofilm reduction in MDR strains of *P. aeruginosa* [95]. A group of scientists formulated a cocktail of novel phages that could lyse planktonic and biofilm cells of *P. aeruginosa*. Furthermore, it is effective in treating acute respiratory infections in mice and bacteremia in *Galleria mellonella* larvae. The phage cocktail demonstrated better biofilm inhibitory activity than that of individual phages, and this activity was independent of multi-drug resistance in *P. aeruginosa* [96].

Zurabov et al. conducted a study to evaluate the effects of bacteriophages with depolymerase activity against biofilms formed by antibiotic-resistant *K. pneumonia*. A cocktail of three bacteriophages, vB_KpnS_FZ10, vB_KpnP_FZ12, and vB_KpnM_FZ14, was prepared, and their antibiofilm activity was tested against the bacterial populations under in vivo and in vitro conditions. Optical microscope biofilm imaging revealed that the antibiofilm activity of the phage cocktails was similar to that of bacteriophage vB_KpnP_FZ12. Similar results were obtained using scanning electron microscopy biofilm imaging. In the bacterial samples treated with the bacteriophage cocktail and vB_KpnP_FZ12, only individual bacterial cells and small aggregates were observed after incubation for 24 h, and subsequent incubation for 24 and 48 h. The experiments demonstrated similar efficiency using both the cocktail and a single phage; however, the use of phage cocktails was recommended to avoid the development of phage resistance and increase the lytic effects by diversifying the number of target pathogens [97].

## 4. Phage-Derived Enzymes

Phage-derived enzymes have been widely investigated as antibiofilm agents and demonstrated efficacy in controlling and removing biofilms [98].

### 4.1. Holin-Endolysin System

Bacteriophages with large genomes, such as bacteriophage λ, use at least two enzymes to induce host lysine, which forms the holin-endolysin system [99].

#### 4.1.1. Holins

**Figure 2:**
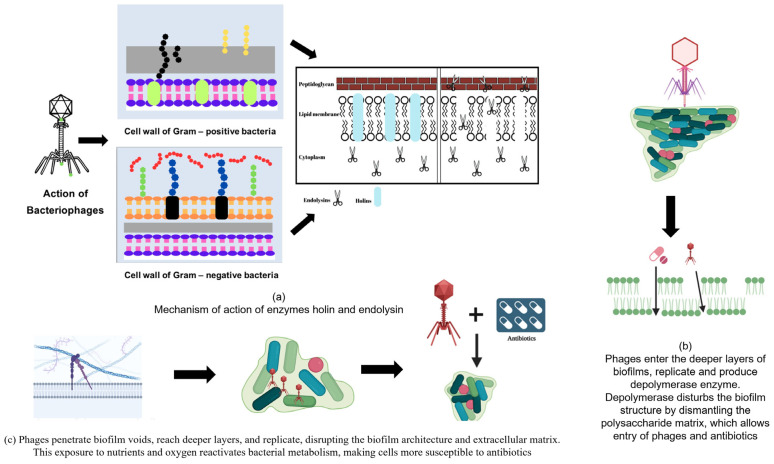
(**a**) Action of Bacteriophage enzymes (Holins and Endolysins) in the degradation of the bacterial cell wall. (**b**) Mechanism of synergistic action between antibiotics and bacteriophages (enzymes such as depolymerase) for biofilm inhibition. (**c**) Mechanism of synergistic action between antibiotics and bacteriophages for biofilm inhibition.

Holins are a group of short hydrophobic polypeptides composed of 130 amino acids encoded in the phage genome. These enzymes can form holes in the cell membrane, allowing other enzymes (endolysins) to enter the cell. They display low sequence similarities between the different proteins. However, they show some similarities in the arrangement of charged polar amino acid residues and their secondary structure. They have a positively charged and hydrophilic C-terminal domain (CTD) and a hydrophobic transmembrane domain (TMD), characterized by the presence of an alpha-helical segment, which is synthesized during the late phase of infection and accumulates in the inner membrane of bacterial cells to form homodimers [100]. Once they reach a critical concentration, they break down the proton motive force (PMF) of the cell, leading to the formation of holes in a process called triggering. Endolysins are released into the periplasmic space by forming holes, allowing them to reach the peptidoglycan layer [101]. Holins form holes of approximately 200–400 nm but differ among different phages. They are localized in the cell membrane owing to the presence of hydrophobic helical domains and therefore lack signal sequences. The modes of action of the holes are illustrated in Figure 2a.

Cahil et al. demonstrated that holins are responsible for controlling the site of bacterial lysis by analyzing S105 lysin using fluorescent video microscopy in lambda phages. This is because holin rafts are generally synthesized at the poles [102]. Based on the size and number of TMDs, holins can be divided into three classes: class 1, 2, and 3 [101].

#### 4.1.2. Role of Lysin in Phage-Mediated Biofilm Control

The bacteriophage lytic enzyme (or endolysin), which is produced during the lytic cycle of double-stranded phages, is vital for the control of biofilms formed by Gram-positive bacteria because it is capable of degrading bacterial peptidoglycan. This peptidoglycan hydrolase cleaves bonds in the bacterial cell wall, ultimately leading to bacterial death [74,103]. Most endolysins have a modular structure with a C-terminal cell wall-binding domain (CBD) linked to one or two N-terminal enzymatically active domains (EAD) by a short and flexible linker region. EAD cleaves the bonds in the peptidoglycan layer and CBD identifies and attaches to epitopes on bacterial cells. These act by disrupting the cell wall and lysing the bacteria at the end of their life cycle. In Gram-positive bacteria, endolysins demonstrate bactericidal activity by degrading peptidoglycans [104]. The cell wall of Gram-negative bacteria is protected by the outer membrane of the lipopolysaccharide; hence, this group of bacteria is partially shielded from the action of endolysins. Certain endolysins that affect Gram-negative bacteria possess globular structures [105]. Endolysins can be divided into different classes based on the type of bonds they cleave [74,106]. Different classes of endolysins have been previously described. Cell wall glycosidase includes endo-β-N-acetylmuramidase (lysozyme), which hydrolyses the β-1,4-glycosidic bonds that exist between N-acetylmuramic acid (MurNAc) and N-acetylglucosamine (GlcNAc). Lytic transglycolase catalyzes the non-hydrolytic cleavage of the N-acetylmuramoyl-β-1,4-N-acetyl glucosamine bond, a glycosidic bond between the alternating MurNAc and GlcNAc disaccharide of the peptidoglycan backbone by an intramolecular cyclization of the N-acetylmuramyl moiety to produce a 1,6-anhydro-N-acetyl-β-D-muramyl (1,6-anhydroMurNAc) product [105,107]. N-acetyl-β-D-glucosaminidase (NAGase) catalyzes the hydrolytic cleavage of the N-acetylglucosaminyl-β-1,4-N-acetylmuramine bond present between the disaccharides [105]. Cell wall amidases include N-acetylmuramoyl-L-alanine amidases, which cleave the bond between sugars and stem peptides. Cell wall peptidases include endopeptidases that cleave the bond between two amino acids of the stem peptide or interpeptide bridges [105]. Exo-β-N-acetylmuramidases catalyze exo-lytic cleavage of β-1,4-MurNAc entities from the non-reducing ends of peptidoglycan chains.

Son et al. demonstrated that the cell wall-degrading enzyme, endolysin SAL-2, isolated from the bacteriophage, SAP-2, possesses substantial biofilm removal potential against *S. aureus* [108]. Another endolysin, LyH5, effectively removed *S. aureus* and *Staphlococcus epidermidis* biofilms in vitro. Additionally, 1–3 log units reduced the cell count in the biofilm upon treatment with LyH5 and persister cells were lysed. Notably, the concentrations below the MIC did not induce biofilm formation [109]. Engineered endolysins have been used to control and eradicate biofilms. LysK (staphylococcal endolysin) was used to derive peptidase CHAP_k_. This engineered enzyme not only prevented biofilm formation but also removed the pre-formed staphylococcal biofilm after 4 h of its application [110]. In another study using engineered lysins, chimeolysin (ClyrR) was tested against penicillin-resistant strains of the dental pathogen, *S. mutans*. ClyR (100 µg/mL) reduced *S. mutans* biofilm by approximately 2 and 3 log under sugar and cariogenic conditions, respectively [111]. The bacteriophage lysin CF-301 effectively disrupted the mature biofilms of *S. aureus*, *Streptococcus pyogenes*, and *Streptococcus agalactiae*. CF-301 effectively removed *S. aureus* biofilms that formed on various surfaces, including catheters. It removed almost all the biofilm cells within 1 h of its application and 100% of the released bacterial population was eliminated by 6 h [112]. The synergistic effect of two lysins, Cpl-711 and PL3, on the inhibition *Streptococcus pneumoniae* biofilm was demonstrated by Vazquez and Garcia [113]. The synergy between the two phage lysins resulted in the use of a reduced amount of enzyme and increased efficacy against the biofilms of *S. pneumoniae*. Sub-MICs of a combination of Cpl-711 and PL3 demonstrated increased killing of free-living cells by 2.4 logs, whereas the synergistic interactions resulted in reduced biofilm biomass by 3.6 logs.

The use of endolysins in Gram-negative bacteria is mainly limited to Gram-positive bacteria because the outer protective membrane of Gram-negative bacteria is believed to be impermeable. To overcome this problem, artilysins have been designed and developed to penetrate protective outer membranes. These modifications of endolysins possess immense antibacterial activity [114]. Antimicrobial peptides help increase the access of endolysins to the peptidoglycan layer. They are effective against both Gram-positive and -negative bacteria. Wang et al. created three antimicrobial-peptide-lysin fusion proteins to identify dual-target antibacterial proteins by fusing sheep myeloid 29 amino acid peptide (SMAP29) to the N-terminal of LysPA26 (an endolysin containing a lysozyme-like domain) using three amino acids as linkers [115]. This fusion improved the antibacterial activity by targeting both the peptidoglycan layer and membrane. Three proteins were generated using flexible linkers: SMAP29-GSA-LysPA26 (AL-3AA), SMAP29-(GSA)3-LysPA26 (AL-9AA), and SMAP29-(GGGGS)3-LysPA26 (AL-15AA). These results indicated that AL-3AA inhibited *P. aeruginosa* PAO1 biofilm formation and eradicated pre-formed biofilms. It can quickly lyse and disintegrate bacteria. It also displayed broad-spectrum antimicrobial activity against Gram-negative bacteria, such as *K. pneumoniae* and *E. coli*. These results indicate that AL-3AA can be developed as a treatment for *P. aeruginosa* biofilms [116].

Recently, their application against Gram-negative bacteria exhibited a substantial increase in efficacy when combined with membrane permeabilizers [103]. Chelators, such as ethylenediaminetetraacetic acid, nitrilotriacetic acid, and sodium hexametaphosphate, which can remove Mg^2+^ and Ca^2+^, can act as effective membrane permeabilizers. Certain polycations, such as polymyxins and their derivatives, can bind to the anionic sites of lipopolysaccharides and render the membrane permeable to drugs [117].

## 5. Engineered Endolysins

### 5.1. Innolysins

Innolysins are fusion products created by merging the receptor-binding protein (RBPs) of bacteriophages with endolysins. This method can be used to specifically target Gram-negative bacteria [105]. It combines the binding capacity of phage RBPs with the enzymatic activity of lysin. RBP mediate adhesion specificity by forming fibers or spikes at the phage tail. Phages recognize their host bacteria by binding to surface receptors, which include lipopolysaccharides or components of bacterial cell walls. Zampara et al. constructed several innolysin molecules by binding RBP Pb5 to the phage T5 endolysin. Ec21 exhibits the highest antibacterial activity. When tested against *E. coli*, a significant reduction was observed in the cell count [118].

### 5.2. Lysocin (Lysin-Bacteriocin)

Lysocins are fusion proteins formed by merging endolysins with bacteriocins (antibacterial proteins). They help transport molecules across the outer membrane (OM translocation). Heselpoth et al. introduced the bacteriocin pyocin S2 (PYS2) from *Pseudomonas aeruginosa*, which was fused with GN4 lysin to produce PyS2-GN4 lysocin. Lysocin allows the translocation of GN4 to the periplasmic space to cleave the peptidoglycan layer, and the PyS2 is responsible for surface receptor binding and translocation. The protein displayed better properties than antibiotics. Lysocins have shown efficacy against biofilms and are not cytotoxic to host cells [119].

### 5.3. Pinholins–Signal Arrest Release (SAR) System

SAR is an evolutionary intermediate lytic system that was originally discovered in the lambda phage 21. S^21^ encodes pinholin and the SAR endolysin (R^21^). Phage 21 pinholin forms approximately 2 nm pores from the heptameric pinholes. Pinholins cause depolarization of the inner membrane by allowing the flow of protons and dissipating the PMF [98]. This results in the release of SAR endolysin into the periplasm and its conversion from an inactive to an enzymatically active state by refolding, leading to the degradation of peptidoglycan. This method is used by approximately 25% of the phages [120].

### 5.4. Spanins

Spanins are a group of bacteriophage-encoded enzymes that act on the outer bacterial membranes. They have not gained as much popularity as endolysin-holin systems, which are capable of disrupting cell walls and causing lysis. In lambda phages, spanins form a heterotetrameric complex composed of two copies of Rz (inner membrane) and Rz1 (outer membrane spanning the periplasmic space). The saponin complex undergoes oligomerization and conformational changes that degrade the PG layer, leading to merging of the inner and outer membranes [120]. In the absence of spanins, bacteriophages use disrupted proteins to degrade the outer membranes [120]. One such example is gp28, which is a cationic antimicrobial peptide [121]. Two major types of spans are identified. These include a unimolecular spanin (u-spanin) and two-component spanin (o-spanin for the outer membrane and i-spanin for the inner membrane) [122].

### 5.5. Virion-Associated Lysins (VAL)/Virion-Associated Peptidoglycan Hydrolase (VAPGH)

VAR and VAPGH are exolysins and consist of muralytic enzymes associated with phage tails. They are active during the attachment and adsorption stages of the lytic cycle and facilitate phage penetration through the bacterial cell wall. They act via a glycosidase or endopeptidase mode of action and reduce the risk of developing resistance owing to the presence of a dual catalytic domain. They rupture the cell wall at specific points, allowing the phages to inject genetic material [123]. They display several beneficial characteristics, such as stability at high temperatures, effective action against drug-resistant bacteria, and modular organization. It displays limited specificity in Gram-positive bacteria; however, broad-spectrum effects have been observed in Gram-negative bacteria [124]. It can be engineered to attach to the CBD of endolysins and used against bacteria. Experiments involving shuffling of the CBD between bacteria have been performed, leading to the formation of chimeric lysins.

### 5.6. Amurins

Amurins are novel direct lysing agents [125]. The term amurin is used to describe antimicrobial lysin proteins that have the potential to disrupt the peptidoglycan layer but do not have muralytic activity, which means that they lack the ability to remove the muramyl group. They are generally produced by single-stranded RNA (ssRNA) or single-stranded DNA bacteriophages with genome sizes smaller than 6 kb [126]. They display broad-spectrum activity against Gram-negative bacteria and their biofilms [74]. They are sometimes referred to as protein antibiotics because their lytic action is similar to that of antibiotics and they are part of a group called single-gene lysis proteins (sgl). Three of the 11 identified sgl proteins are protein antibiotics. This interfered with the steps involved in providing lipid II to the peptidoglycan layer. The sgl system consists of a passive method that does not degrade the cell wall; instead, it induces lysis of the host cell [127].

There are three major examples of Type I sgl systems. An important example is E protein, a 91-amino-acid residue protein encoded by the *E* gene, which is embedded out of frame within the *D* gene of the φX174 bacteriophage belonging to the *Microviridae* family. Structurally, E protein comprises a conserved N-terminal transmembrane helix and an extended cytoplasmic C-terminal region. It facilitates host cell lysis by inhibiting peptidoglycan cell-wall synthesis, thereby compromising bacterial cell integrity.

The mechanism of action of the E protein involves targeting the bacterial enzyme MraY transferase, which belongs to the polyprenyl phosphate–N-acetylhexosamine-1-phosphate transferase superfamily. MRAY catalyzes a crucial step in peptidoglycan biosynthesis by transferring the phospho-N-acetylmuramoyl-pentapeptide motif to an undecaprenyl phosphate carrier lipid, resulting in the formation of lipid I, the first membrane-bound intermediate in the peptidoglycan synthesis pathway. Lipid I resides on the cytoplasmic side of the bacterial membrane and is an essential precursor for cell wall assembly. When the E protein binds to MraY, it obstructs the active or substrate-binding site of the enzyme, thereby inhibiting lipid I formation and ultimately preventing peptidoglycan synthesis, which leads to bacterial lysis [99,128,129,130].

Additionally, the sensitive to lysin D (SlyD) protein is required for the stable accumulation of the E protein. SlyD functions as a bacterial chaperone, containing both a peptidyl-prolyl cis-trans isomerase and chaperone domain. The interaction between the E protein, MraY, and SlyD results in the formation of a transmembrane YES complex that stabilizes the inhibitory interaction. Inhibition of MraY enzymatic activity by the E protein leads to the loss of cell wall synthesis and subsequent lysis of the host bacterium. Mutations in MraY that confer resistance to E protein-mediated lysis have been identified in some bacterial strains, highlighting the specificity of this interaction.

Type I Sgl proteins include the A2 and Lys proteins. The A2 protein, a 460-amino-acid component encoded by the *L* gene of the Qβ bacteriophage (family Alloleviviridae), occurs at one copy per virion. It induces host cell lysis by binding to MurA, a bacterial enzyme essential for cell-wall biosynthesis, thereby inhibiting the first committed step in peptidoglycan synthesis [99]. Another Type I Sgl, the Lys protein from coliphage M, targets MurJ, a lipid flippase responsible for translocating lipid II, a peptidoglycan precursor, across the membrane. LysM inhibits MurJ activity by locking it to a specific conformation, thereby blocking peptidoglycan synthesis, and leading to cell lysis [99,131].

The Type II Sgl system is represented by the L protein, a 75-amino-acid residue protein encoded by the *L* gene of the MS2 bacteriophage, which belongs to the *Leviviridae* family and possesses a ssRNA genome. Structurally, the L protein comprises a hydrophilic N-terminal domain rich in several basic amino acids and a hydrophobic CTD. Although the detailed mechanism of action of the L protein has not been fully elucidated, it is known to induce lysis in *E. coli* and hypothesized to directly target host proteins, possibly through interactions with DnaJ, a bacterial chaperone protein [131,132].

## 6. Role of Phage-Encoded Depolymerases in Biofilm Control

Another phage-derived enzyme that has been exploited for biofilm control is phage depolymerase. Bacteriophages whose genome encodes EPS depolymerase can use these polysaccharides as primary receptors and cleave the bonds until they reach the cell membrane. This may have contributed to the biofilm degradation [97].

Generally, depolymerases are encoded as a part of the phage structure. The activity of the depolymerase is shown in Figure 2b. Depolymerases are capable of acting on components, such as capsular polysaccharide, lipopolysaccharide, and EPSs of the biofilm that expose the bacterial cell surface receptors for bacteriophages to attach to and act on bacterial cells. These are non-lytic enzymes, that is, the enzymes do not cause cell lysis but support the host immune system and antibiotics by allowing them to target cells effectively [133]. Depolymerases can be classified into hydrolases and lyases based on their mechanisms of action. Hydrolases cleave their substrates in a hydrolytic manner, whereas lyases perform depolymerization without the use of water molecules, that is non-hydrolytically [77,78].

Depolymerase Dpo7 considerably decreased biofilm formation (>90%) in polysaccharide-producing strains, although it was ineffective against polysaccharide-independent biofilms. The Dpo7 protein possesses a pectin lyase domain characterized by a right-handed β-helix structure. Proteins featuring such repetitions typically use polysaccharides as substrates and hence eliminates polysaccharides [134].

Depolymerase Dpo7 reduces the EPS by 31–75% in different strains of *S. epidermidis* as compared to the untreated control. Furthermore, the coating of polystyrene surfaces with Dpo7 caused a 53–85% decrease in the biofilm biomass of the test strains [134]. A phage belonging to the Myoviridae family, encoding functionally active Dpo42, was tested for its antibiofilm potential. Dpo42 caused dose-dependent degradation of capsular EPSs and reduced biofilm formation. The highest biofilm inhibition was recorded upon treatment of *E. coli* biofilm cells with 25 µg/mL of Dpo42 [135]. Bacteriophage Petty, a 40,431 bp ϕKMV-like phage possessing a gene of depolymerase Dpo1 demonstrated reduced EPS and biofilm formation in MDR *Acinetobacter nosocomialis* and *Acinetobacter baumanii.* Dpo1 significantly degrades capsular EPSs, which are virulence factors that substantially contribute to the formation and biofilm maturation [136]. In another study, a novel lytic phage, IME180, possessing genes encoding a functional depolymerase, was evaluated against *P. aeruginosa* biofilms. This enzyme caused a considerable reduction in EPS production and inhibited biofilm formation in *P. aeruginosa*. Additionally, at 30 µg/mL concentration, the depolymerase could disrupt pre-formed biofilms, although the removal was not absolute [137]. In a recent study, Chen et al. isolated the lytic phage, PHB19, and identified a depolymerase (Dep6) in its tail spike protein. Both the phage and Dep6 reduced the biofilm biomass of STEC. Dep6 decreased 24 and 48 h biofilm by 29% and 54%, respectively, compared to the untreated control [72].

## 7. Combination Therapy with Phage and Antibiotics

The use of phages in combination with conventional antibiotics was investigated. Previous investigations have suggested that sub-inhibitory concentrations of antibiotics could prove effective in improving the production and activity of virulent phages, known as phage-antibiotic synergy [82,138]. The synergistic action of the bacteriophages and antibiotics is shown in Figure 2c.

In a previous study, phages were engineered to overexpress proteins and target a network of genes that escape the action of antibiotics. The engineered phage, in synergy with ofloxacin, enhanced the killing of *E. coli* biofilm cells by 1.5 and 2 orders of magnitude [139]. Bedi et al. assessed the biofilm inhibitory potential of bacteriophages alone or in combination with amoxicillin against *K. pneumoniae* B50555 strain. The phage and antibiotic (amoxicillin) were effective in reducing the biomass of 1-day old biofilm at the MIC, i.e., 256 µg/mL but insignificant reduction was recorded with 8-day old mature biofilm. Notably, the exposure of *K. pneumoniae* biofilms to a combination of phages and amoxicillin resulted in significantly reduced biofilm biomass [140]. In a study conducted on the inhibition of *S. aureus* biofilms by a phage and three antibiotics, phage SAP-26 alone could reduce biofilm formation by 28%. Three antibiotics, namely, rifampicin, azithromycin, and vancomycin, demonstrated 40, 25, and 17% killing of biofilm bacteria, respectively. Significantly enhanced synergism was recorded when *S. aureus* biofilms were exposed to a combination of phages and rifampicin, and approximately 65% reduction in the biofilm biomass was recorded. In another study, the combination of SAP-26 and azithromycin resulted in a 60% reduction, whereas SAP-26 and vancomycin decreased biofilm cells by 40% [141]. Yilmaz et al. evaluated the effect of a combination of phages and antibiotics on the biofilms of MRSA and *P. aeruginosa* using an implant-related infection model [142]. In the case of MRSA strains, although both the phage and antibiotics demonstrated bactericidal action, biofilm formation was inhibited only by the combination of the phage and antibiotic (teicoplanin). The combination of phages and antibiotics (imipenem, amikacin, and cilastatin) had an effect on *P. aeruginosa* biofilms; however, this effect was not significant. Overall, the group observed that the bacteriophage, in synergy with antibiotics, reduced biofilm formation in both test bacteria; however, the effect was more pronounced on MRSA than that on *P. aeruginosa*. In vitro biofilms of *P. aeruginosa* were exposed to phages and antibiotics separately as well as in combination. The modest efficacy was recorded when the phages and antibiotics were administered individually. However, the synergistic interaction of phage and antibiotics accounted for increased efficacy (cell densities were reduced by 3 and 5 orders of magnitude compared with that of the control) against the *P. aeruginosa* biofilm growing on layers of cultured epithelial cells [143]. In another investigation conducted on biofilms formed by clinical drug-resistant isolates of *P. aeruginosa*, a combination of phages and antibiotics (ciprofloxacin) proved to be effective. The combination of the PEV20 phage and ciprofloxacin significantly increased biofilm removal. Furthermore, this combination protected lung epithelial and fibroblast cells from *P. aeruginosa* infection and promoted normal cell growth. The synergy between phage PEV20 and ciprofloxacin is dependent on the phage resistance profile of the target bacteria [144]. In a recent report, the lytic phage KP34, a depolymerase, a non-depolymerase bearing phage KP15, and ciprofloxacin were used separately and in combination against a MDR *K. pneumoniae* biofilm model. The results of the antibiofilm assay revealed that two combinations demonstrated the highest efficacy: phage KP34 in combination with ciprofloxacin, and KP34 and KP15 with ciprofloxacin. The aforementioned combination resulted in 4.1 log reduction in CFU and 83.5% drop in the biofilm biomass [145].

The synergistic activity of phages and antibiotics can be attributed to the bactericidal action of phages, which makes biofilms more susceptible to the action of antibiotics. Phages interact with biofilms by attaching to specific receptors, lysing and entering cells, and disrupting the biofilm. This may be due to depolymerase activity, which dismantle the matrix composed of polysaccharides and allow the easy passage and spread of phages. This passage, created by the phages passing through the biofilm, aids in the diffusion of higher concentrations of antibiotics inside the biofilm compared with that of the untreated intact biofilms [140,146,147]. In another study, bacteriophages and antibiotics degraded the biofilm of *P. aeruginosa*. No depolymerase was detected in the vB_PaM_EPA1 phage; therefore, it was suggested that the phage would move through the biofilm void spaces and reach the bottom layers. Biofilm architecture is disturbed by the replication of phages in the deep layers of the biofilm, which increases the efficacy of antibiotics [148]. The combination of PEV20 phage and ciprofloxacin significantly increased the removal of biofilm compared with that using the single treatment with the phage or ciprofloxacin. This is due to bacteriophage activity that disturbs the outer matrix, exposing the biofilm cells to nutrients and oxygen. Metabolically active cells are susceptible to antibiotics and phages [144].

## 8. Mechanism of Action

**Figure 3:**
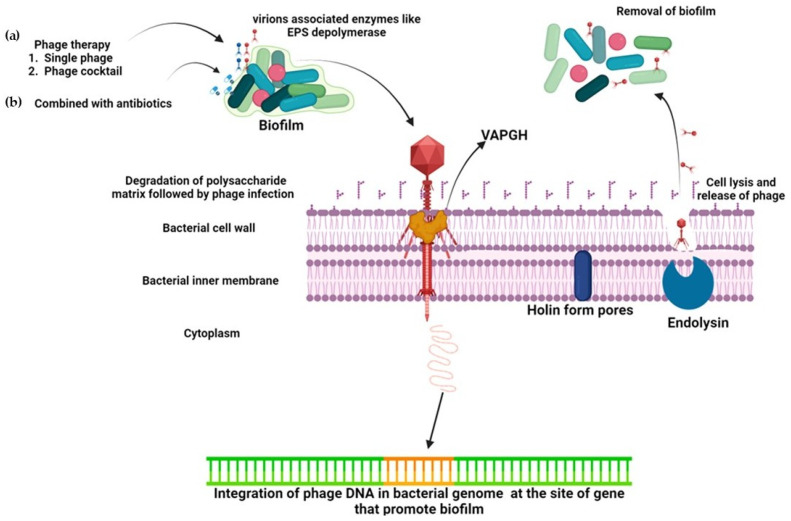
The inhibitory action of bacteriophages against biofilms, (**a**) phage therapy alone (**b**) with a combination of antibiotics.

Several mechanisms of biofilm removal by phages have been explored. The different mechanisms of action of bacteriophages on biofilms are shown in Figure 3. Lysogenic phages integrate into the host (bacterial) genome and impair biofilm formation in the host bacteria [149]. For instance, the integration of phage Bxb1 into the genome of *Mycobacterium smegmatis* results in the inactivation of the *groEL1* gene, which helps in the maturation of biofilms, thereby impairing biofilm maturation and removal [150]. Another mechanism applied by phages to control biofilm formation is the production of lyases and lytic proteins that lyse bacterial cell walls. Engineered bacteriophage expressing enzyme DspB that damages β-1,6-N-acetyl-D-glucosamine was designed to explore its action on biofilms. β-1,6-N-acetyl-D-glucosamine is vital during the adhesion stage of the biofilm formation and when DspB is released into the environment upon the lysis of the host, it accounts for enhanced biofilm eradication. Hence, DspB is effective during the initial stages (adhesion) of biofilm formation as well as against mature biofilms and capable of removing 99.997% of the biofilm [139]. Some phages are equipped with lytic enzymes (VAPGHs). These enzymes produce a hole in the host cell wall, through which genetic material reaches the cytoplasm [151].

In addition to producing lytic enzymes and proteins, phages express a variety of enzymes that act on extra polymeric materials and proteins that form the matrix and encapsulate bacteria in the biofilm. The action of phage enzymes exposes cells within the biofilm matrix, making them more susceptible and prone to destruction.

Table 1 lists different bacteriophages and their products which have been experimentally tested for their efficacy against the biofilms of different bacteria.

## 9. Resistance of Bacterial Biofilms Against Phage Infections

Several mechanisms in bacteria and their biofilms that make them efficient in resisting phage infections have been demonstrated. Various mechanisms have evolved to inhibit infection at different stages. Abortive infective (Abi) systems, restriction modification (RM system), superinfection exclusion, surface modification, and clustered regularly interspaced short palindromic repeats (CRISPR-Cas) are processes known to help in the defense against phages [177].

The Abi system is activated at later stages of the infection cycle. It causes phage-infected bacterial cells to commit suicide before the completion of the phage replication cycle, thereby preventing the spread of phages to neighboring cells. Thus, the entire bacterial population is protected from damage by the prevention of phage proliferation. Therefore, this is considered an altruistic trait. Cyclic oligonucleotide-based antibody signaling systems are a large family of Abi systems that have recently been discovered. Using this method, phage infection can activate the DncV protein, which produces cGMP-AMP. When cGAMP accumulates, it can activate the phospholipase, CapV, which can degrade the inner cell membrane, leading to cell lysis and death.

RM systems are composed of restriction endonucleases and methyltransferases that help identify foreign DNA that lack certain modifications at the recognition site. This method avoids the destruction of the self-genome by methylation of recognition sites.

The CRISPR-Cas system protects bacteria from plasmids and phages by identifying and cleaving foreign nucleic acids specified by the spacer sequences. Phage-infected bacterial cells can quickly become resistant to infections by related phages and defend themselves by preventing the entry of phage nucleic acids. This method involves the exclusion of superinfection. The surface modification of bacteria prevents phage infection by blocking the initial adsorption of phages.

The defense island system associated with restriction-modification (DISARM) is a novel method that restricts incoming phages. Several genes associated with bacterial and archaeal defense are physically clustered in a genomic locus known as “defense islands.” DISARM is composed of five genes, including a DNA methylase and other genes annotated as the helicase domain, phospholipase-D-D domain, DUF1998 domain, and a gene of unknown function [178].

## 10. Limitations of Phage Therapy

In addition to the efficacy of bacteriophages, safety issues must be evaluated. Phages with complete molecular characterization and whole-genome sequencing should be considered for future use to bypass the existence of virulence and drug-resistance genes. The high specificity of bacteriophages limits their use as therapeutics; however, the application of a cocktail of phages and polyvalent phages possessing a broad-spectrum host range can overcome the problem of specificity. Another issue is the use of engineered phages; consumers have shown reluctance due to genetic manipulation [179].

Large-scale production using inexpensive protocols is another area that requires further attention. Methods for propagation and purification require optimization. Although the methods used in laboratories are standardized and optimized, they are not easy to scale up and are not cost-effective for large-scale production. In addition to the production of phages and their derived proteins, parameters, such as proper formulation, stability in non-refrigerated environments, and lytic activity under normal conditions, must also be considered [27].

The thermostability of phage-derived proteins is another area of concern before they can be used as therapeutics. The thermolabile nature of endolysins is well documented, but heat-stable proteins have only recently been discovered. Novel thermostable lysins, Lys68, from *Salmonella* phages, phi68 and Ph2119, have been described [165,180]. However, there are problems associated with endolysin treatment. Endolysin-based drugs are commonly administered orally, making endolysins susceptible to degradation by protein-degrading enzymes. The nasal route for drug delivery can be considered; however, it has its own set of restrictions owing to the limited availability of an absorbing surface in the nasal cavity and the large size of endolysins, which makes crossing through the nasal mucosa a challenge. The delivery of endolysin can be achieved through intravenous injection; however, its invasive nature and the risk of secondary bacterial infections at the site of injection are major issues that require attention. Overcoming this limitation requires further engineering of the endolysins [123].

## 11. Conclusions

Biofilm formation significantly increases bacterial virulence. Several methods have been applied to combat biofilms, which are important in industrial and medical settings. Phage therapy is an attractive option for the prevention and control of biofilms and related infections. Phage infection of biofilms is efficient when cells are in close proximity. However, biofilms are resistant to phages because of their dense matrix, low metabolic state, and enhanced proliferation of phage-resistant variants. Phage cocktails offer several benefits over individual phages for overcoming resistance. Moreover, phage-based enzymes, such as endolysins, holins, depolymerases, and engineered enzymes offer potentially effective alternatives. A combination of antibiotics and phages may be effective in reducing biofilms. However, there is a need to understand and expand the scope of the application of phages in mixtures and engineered phage transformations. A thorough evaluation of the application of phages needs to be carried out, as phage therapy could prove to be a powerful tool against persistent biofilm-based infections.

## Figures and Tables

**Table 1:** Application of bacteriophages and combination therapies for biofilm inhibition.

	Biofilm Type	Efficacy	Reference
Phage			
Phage P100	*L. monocytogenes*	Reduced cell counts from 3.5 to 5.4 log units/cm^2^	[84]
		Decreased biofilm cells to undetectable levels after 48 h	[152]
Phage KH1	*E. coli* O157:H7	1.2 log units per coupon reduction after 4 days application	[153,154]
Phage SAP-26	*S. aureus*	28% reduction in bacterial biomass	[141]
Phages ISP, Romulus and Remus	*S. aureus*	37.8%, 34.4%, and 60% reduction in biofilm by Phages ISP, Romulus, and Remus, respectively	[155]
Cocktail of phages			
Phage cocktail containing 39APmC32, 65APm2833 and 72APm5211	*P. mirabilis*	The phage cocktail showed antibiofilm activity against 2–3 strains more than the activity of single phages without hindering the activity of each other	[156]
Phage K and phage derivatives	*S. aureus*	Complete inhibition of biofilm formation	[91]
Phage K and DRA88	*S. aureus*	Complete biomass inhibition after 48 h of phage application	[92]
Phages LiMN4L, LiMN4p and LiMN17	*L. monocytogenes*	Within 75 min biofilm cells reduced to undetectable levels	[80,85]
Phage CP8 and CP30	*C. jejuni*	1–3 logs unit/cm^2^ decrease in biofilm cell counts	[157]
Phages of Herelleviridae family (B2-102, O1-102, T2-102, and O2-92)	*S. aureus*	Log reduction in viable cell counts ranged from 3.1 to 4.2 cfu/g	[158]
01 BEC8 (Phage cocktail)	*E. coli O157:H7*	Significant biofilm reduction after 1 h of phage treatment	[159,160]
Cocktail of 3 phages (L8, SAEN098P01, and SAEN098P03)	*Salmonella*	It was highly effective against several serovars of the bacteria	[161]
Phage protein			
Endolysin (Phage phi11)	*S. aureus*	Complete of inhibition of *S. aureus* biomass	[162,163]
Endolysin SAL-2	*S. aureus*	Reduced biomass after 2 h of application	[80,108]
Endolysin LysH5	*S. aureus*	Biofilm cell counts reduced by 1–3 log units	[109]
Domain CHAP_k_ derived from endolysin LysK	*S. aureus*	Complete biofilm inhibition was recorded	[110]
Chimeric lysin ClyH	*S. aureus*	More than 60% reduction in biomass	[164]
Endolysin Lys68	*S. typhimurium*	1 log unit reduction in biofilm biomass	[165]
Depolymerases			
Exoplysaccharide depolymerase Dpo7	*S. aureus*	Inhibition of biofilm polysaccharide matrix	[134]
Depolymerase Dpo42	*E. coli*	Reduction in capsular exopolysaccharides and biofilm in a dose dependent manner	[135]
Depolymerase Dpo1	*A. nosocomialis, A. baumanii*	Inhibition of capsular exopolysaccharides and biofilm	[136]
phage IME180 depolymerase	*P. aeruginosa*	Inhibition of pre-formed biofilm at 30 µg/ml	[137]
Dep6	*Shiga toxin producing E. coli (STEC)*	Reduced 24 h and 48 h biofilm by 29% and 54%	[72]
Combined therapy with antibiotics			
Depolymerase encoded by phage 168 along with polymyxin B	*Carbapenem-Resistant K. pneumoniae*	Disruption of biofilm was done by depolymerase and the polymyxin exerted its bactericidal effects. They showed symbiotic action and bacterial load reduced	[166]
Depolymerase from phage KPO1K2 and ciprofloxacin	*K. pneumoniae strain B5055*	Increased biofilm inhibition and removal	[67,167,168]
Phage cocktail with ciproflaxin or meropenem (2.5 X MIC)	*P. aeruginosa*	It inhibited the regrowth of phage-resistant mutants	[169]
Phage SAP-26 and rifampicin/azithromycin/vancomycin	*S. aureus*	Disruption of biofilm biomass matrix and 4-log reduction	[141,170]
Phage PSPS (Pbunavirus) with ciproflaxin	*P. aeruginosa PAO1 biofilms*	The combination resulted in decrease in biomass reduction by 24.7%. Up to 29.7% decrease in biomass of biofilm	[171]
T4 phage and cefotaxime	*E. coli*	Synergistic action resulted in reduced MBEC of cefotaxime by 2–8 folds against *E. coli*	[172]
T4 phage and tobramycin	*E. coli*	Synergistic action resulted in approx. 99.99% reduction	[173]
Depolymerase from phage KPO1K2 and gentamicin	*K. pneumoniae B5055*	Reduced biofilm biomass counts of young biofilms (up to 4 days)	[174]
Phage vB_PmiS_TH and ampicillin	*P. mirabilis*	Highest biofilm removal at 24 h	[147]
PT-bacteriophages and ciprofloxacin	*P. aeruginosa PS573*	Reduced bacterial load by ≥50%	[175]
Depolymerase Dep42 from phage SH-KP15226 and polymyxin	*K. pneumoniae 2226*	Decreased bacterial counts	[176]
PEV20 phage and ciprofloxacin	*P. aeruginosa*	Increased biofilm removal	[144]
Depolymerase KP34p57 from phage KP34 and ciprofloxacin	*K. pneumoniae 77*	Diminished bacterial colony counts	[145]
Phage M1 and ceftazidime and avibactam	*K. pneumoniae*	Decreased bacterial counts in mature biofilms	[81]

## Data Availability

No new data were created or analyzed in this study.
